# Osmolarity‐Induced Altered Intracellular Molecular Crowding Drives Osteoarthritis Pathology

**DOI:** 10.1002/advs.202306722

**Published:** 2024-01-11

**Authors:** Kannan Govindaraj, Marieke Meteling, Jeroen van Rooij, Malin Becker, Andre J. van Wijnen, Jeroen J. J. P. van den Beucken, Yolande F. M. Ramos, Joyce van Meurs, Janine N. Post, Jeroen Leijten

**Affiliations:** ^1^ Department of Developmental Bioengineering Faculty of Science and Technology, Technical Medical Centre University of Twente Drienerlolaan 5 Enschede 7522NB The Netherlands; ^2^ Department of Internal Medicine Erasmus MC Dr. Molewaterplein 40 Rotterdam 3015GD The Netherlands; ^3^ Department of Biochemistry University of Vermont Burlington Vermont VT 05405 USA; ^4^ Dentistry – Regenerative Biomaterials Radboudumc Ph van Leijdenlaan 25 Nijmegen 6525EX The Netherlands; ^5^ Department of Biomedical Data Sciences Section Molecular Epidemiology LUMC Einthovenweg 20 Leiden 2333 ZC The Netherlands; ^6^ Department of Orthopedics & Sports Medicine Erasmus MC Dr. Molewaterplein 40 Rotterdam 3015GD The Netherlands

**Keywords:** cell volume, intracellular molecular crowding, osteoarthritis, pathology

## Abstract

Osteoarthritis (OA) is a multifactorial degenerative joint disease of which the underlying mechanisms are yet to be fully understood. At the molecular level, multiple factors including altered signaling pathways, epigenetics, metabolic imbalance, extracellular matrix degradation, production of matrix metalloproteinases, and inflammatory cytokines, are known to play a detrimental role in OA. However, these factors do not initiate OA, but are mediators or consequences of the disease, while many other factors causing the etiology of OA are still unknown. Here, it is revealed that microenvironmental osmolarity can induce and reverse osteoarthritis‐related behavior of chondrocytes via altered intracellular molecular crowding, which represents a previously unknown mechanism underlying OA pathophysiology. Decreased intracellular crowding is associated with increased sensitivity to proinflammatory triggers and decreased responsiveness to anabolic stimuli. OA‐induced lowered intracellular molecular crowding could be renormalized via exposure to higher extracellular osmolarity such as those found in healthy joints, which reverse OA chondrocyte's sensitivity to catabolic stimuli as well as its glycolytic metabolism.

## Graphical abstract

Osteoarthritis alteration of joint osmolarity is known as consequence of this joint disease, but its role has remained largely unknown. It is demonstrated that healthy osmotic levels sensitize chondrocyte to regeneration and desensitize to inflammation, while an osteoarthritic osmotic level has the opposite effect. Osmolarity regulates pathophysiological effects by altering intracellular molecular crowding, which represents a novel mechanism for therapeutic targeting.

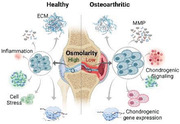

## 1 Introduction

Osteoarthritis (OA) is a complex disease and its pathophysiology is yet to be fully understood.^[^ ^1^ ^]^ Although a lower joint osmolarity has long been recognized as a consequence of OA,^[^ ^2^ ^]^ its role as a potential driving force behind the disease has remained largely uninvestigated. In contrast, for other diseases such as Alzheimer's disease,^[^ ^3^ ^]^ diabetes,^[^ ^4^ ^]^ and sickle cell anemia,^[^ ^5^ ^]^ osmolarity has been identified as an integral part of the etiology in recent years. Regardless, chondrocytes are known to dynamically adjust their volume according to their microenvironmental osmolarity such as those presented by the synovial fluid.^[^ ^6^ ^]^ The osmolarity of synovial fluid and cartilage tissue are known to be lowered in OA (≈300 mOsm) as compared to that found in healthy (≈400 mOsm) joints,^[^ ^2^ ^]^ which results in higher chondrocyte volumes.^[^ ^6^, ^7^ ^]^ A recent study has shown that chondrocytes cultured in unnaturally high osmolarities (e.g., >420 mOsm) decreased inflammatory signaling and increased responsiveness to growth factors.^[^ ^8^ ^]^ Osmolarity also regulates the homeostasis of ECM production, which is essential for smooth joint function.^[^ ^9^ ^]^ Irrigating articular joints with high osmolarity solutions has indeed been suggested to offer chondroprotective properties.^[^ ^10^ ^]^ Despite the importance of osmolarity on chondrocyte homeostasis and its potential role in OA progression, the mechanisms via which osmolarity orchestrates these important processes have remained virtually unknown.

Intracellular molecular crowding dictates the existence, function, and efficiency of virtually all cellular processes.^[^ ^11^ ^]^ Increased molecular crowding promotes protein‐protein interactions, transcription factor activity, and DNA interactions.^[^ ^12^ ^]^ Crowding also affects the stability of protein structures and enzymatic reaction rates. Molecular crowding thus contributes to the regulation of gene expression and functional performance of mRNA, miRNA, and proteins. Importantly, signaling cascades can be distinctly affected by molecular crowding, i.e., anabolic and catabolic signaling pathways can be differently affected by an identical change in molecular crowding.^[^ ^12^, ^13^ ^]^ Molecular crowding is known to affect chondrogenic behavior, for example, it precedes chondrogenic differentiation of stem/progenitor cells.^[^ ^14^ ^]^ Moreover, environmental factors, such as osmolarity^[^ ^15^ ^]^ and matrix stiffness,^[^ ^16^ ^]^ control the level of intracellular molecular crowding, and these environmental factors are known to be affected in osteoarthritis.^[^ ^2^, ^17^ ^]^ However, whether intracellular molecular crowding of chondrocytes is affected by OA, or what the functional consequences are of altered intracellular molecular crowding in chondrocytes has never been studied.

We hypothesized that OA‐induced changes in osmolarity alter the intracellular molecular crowding, which programs the chondrocyte's functional behavior towards an OA‐like phenotype. We therefore have investigated the role of osmolarity and subsequent alteration of intracellular molecular crowding on chondrocyte behavior. We demonstrate that intracellular molecular crowding regulates the chondrocytes’ sensitivity and responsivity to anabolic and catabolic triggers, effectively allowing for a shift towards OA‐like behavior, which at least in part was mediated by altered intracellular molecular crowding.

## 2 Results

### 2.1 OA Increases hPC Cell Size and Volume Leading to Lower Intracellular Molecular Crowding

**Figure 1:**
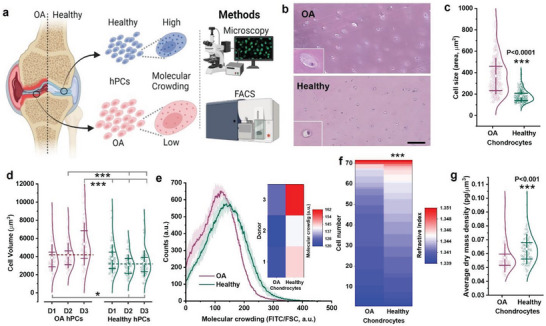
Chondrocyte cell size and volume is inherently higher and that leads to lower molecular crowding in OA hPCs. a) Schematic diagram showing cell size difference between OA and healthy hPCs and the methods used to investigate the cells size. b) Histology sections of OA and healthy cartilage tissue stained with H&E staining, scale bar 100 µm. c) Violin plot showing cell size as measured from the histology sections, *n* = 300 cells. d) Violin plot showing cell volume as measured from live OA and healthy hPCs, *n* > 50 cells, per donor, 3 donors per condition. e) Changes in molecular crowding between live OA and healthy hPCs as measured by flow cytometry. Inlet: Heatmap showing molecular crowding at the individual donor level, *n* = 3 donors. f) Heat map showing the refractive index between live OA and healthy hPCs in iso‐osmotic media (330 mOsm) as measured by holotomography microscopy, 3 donors, *n* > 70 cells. g) Average dry‐mass density of OA and healthy hPCs cultured in iso‐osmotic (330 mOsm) medium for 24 h. Measured using holo‐tomography microscopy, 3 donors, *n* > 70 cells, per condition. Statistics: c,d) Mann‐Whitney U‐test, *p < 0.05, ***p < 0.001, f,g) t‐test, unpaired, ***p < 0.001, c,d,g) whiskers indicate standard deviation (SD) and the box indicate 25%, 75% percentile, e) shades indicate SD.

We hypothesized that the intracellular crowding of chondrocytes was decreased during OA due to the decreased osmolarity that causes an increase in cell size (**Figure** **1**A). To test this hypothesis, we investigated histological sections of healthy cartilage and OA cartilage to confirm that chondrocyte cell size was indeed increased following onset of OA (Figure 1B). In addition, quantitative image analysis demonstrated that the size of chondrocytes was significantly higher in OA cartilage tissue than that of chondrocytes in healthy cartilage (Figure 1C). To confirm that this difference was maintained during in vitro experiments, hPCs were isolated from OA or healthy knee joints and analyzed for cellular volume after being cultured to passage 2 being in iso‐osmotic medium (330 mOsm). This revealed that the observed in vivo differences were also maintained in an in vitro setting, with healthy hPCs having a significantly lower cellular volume (3414 ± 1478µm^3^) than OA hPCs (≈4542 ± 2012 µm^3^, *p* < 0.001; Figure 1D).

High‐throughput flow cytometry measurements based on forward scatter and intensity of total cellular proteins (FITC staining) corroborated that the increase in cellular volume of OA hPCs consistently correlated with a reduction in intracellular molecular crowding (Figure 1E). This observation was further confirmed by holotomography microscopy, which demonstrated that chondrocytes isolated from healthy cartilage possessed a higher refractive index (Figure 1F) and thus were characterized by a higher cell dry‐mass density of 0.0633 ± 0.011 pg µm^−3^ compared to 0.0563 ± 0.010 pg µm^−3^ (*p* < 0.001) for OA chondrocytes (Figure 1G). Together, these data suggest that primary human OA chondrocytes are larger in size and volume, which leads to lower intracellular molecular crowding compared to healthy chondrocytes.

### 2.2 Extracellular Osmolarity Is Higher in Healthy Articular Tissue Compared to OA Articular Tissue

**Figure 2:**
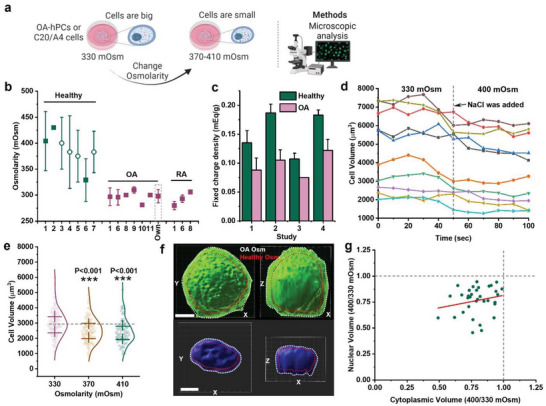
Extracellular osmolarity controls cell size and volume. a) Schematic diagram showing that the cell size can be controlled through osmolarity and the methods used to investigate the cells size. b) Literature reports on osmolarities between healthy and diseased joints (Study 1,^[^ ^2^ ^]^ 2,^[^ ^21^ ^]^ 3,^[^ ^22^ ^]^ 4,^[^ ^23^ ^]^ 5,^[^ ^19e^ ^]^ 6,^[^ ^24^ ^]^ 7,^[^ ^25^ ^]^ 8,^[^ ^26^ ^]^ 9,^[^ ^27^ ^]^ 10^[^ ^28^ ^]^ and 11^[^ ^17b^ ^]^ and our [own] measurements of osmolarity of synovial fluid from OA patients); Filled squares and open circles indicate osmolarity measurements from synovial fluid and cartilage tissue, respectively. c) Fixed charge density measurements of healthy and OA cartilage tissue (Study 1,^[^ ^19c^ ^]^ 2,^[^ ^19d^ ^]^ 3,^[^ ^19b^ ^]^ 4^[^ ^19a^ ^]^). d) Timelapse imaging of chondrocytes cell size change between 330 and 400 mOsm, *n* = 10 cells. e) Change in C20/A4 cell size among 330, 370 and 410 mOsm (*n* > 100 cells, per condition). f) 3D confocal image shows the changes to cell and nuclear size as a cell is exposed to OA or healthy‐like osmolarity, scale bar 5 µm. g) A correlation graph shows the ratio of cytoplasmic and nuclear volume in OA hPCs as they were exposed to OA or healthy‐like osmolarity, *n* = 46. Statistics: e) Mann‐Whitney U‐test, ***p < 0.001, error bars/whiskers in b and e indicate SD and box in e indicate 25%, 75% percentile.

Extracellular osmolarity is a key factor for the regulation of a cell's volume and size (**Figure** **2**A).^[^ ^18^ ^]^ We performed a comparative literature search, which revealed that the osmolarity of synovial fluid and extracellular articular space is consistently higher in healthy joints (≈400 mOsm) compared to joints diagnosed with OA and rheumatoid arthritis (≈300 mOsm; Figure 2B and Table S1, Supporting Information).^[^ ^2^, ^10^, ^17^ ^]^ Our measurements of synovial fluid osmolarity of 16 different human joints suffering from OA confirmed that the osmolarity became as low as 298 ± 13 mOsm (Figure 2B and Table S2, Supporting Information). Along with synovial fluid osmolarity, fixed charge density of cartilage tissue, which in cartilage is dictated by proteoglycans that control local tissue osmolarity by attracting positively charged counter‐ions following the Donnan equilibrium, is also known to consistently decrease in OA cartilage (Figure 2C).^[^ ^7^, ^19^ ^]^ Moreover, several studies have reported that chondrocytes exposed to hyper‐osmotic medium or irrigation solution (>380 mOsm) exhibit higher chondro‐protective features, such as increased ECM production, higher cell viability, and lower postoperative pain in patients, as compared to chondrocytes exposed to hypo‐osmolar (<300 mOsm) solutions (Table S3, Supporting Information).^[^ ^20^ ^]^ Therefore, we investigated the effect of changing the osmotic level from that found in OA cartilage to the level found in healthy cartilage on the size and volume of OA hPCs and C20/A4 chondrocytes. Time‐lapse imaging of suspended OA hPCs showed that an increase in extracellular osmolarity to 400 mOsm resulted in an instant decrease (<10 s) in cell volume by 19 ± 5% (Figure 2D). Similarly, raising the extracellular osmolarity of C20/A4 cells from 330 mOsm to 370 and 410 mOsm resulted in a progressive decrease in cell volume, from 2956 ± 833 µm^3^ for 330 mOsm, to 2516 ± 768 µm^3^ (down by 15%) for 370 mOsm, and 2405 ± 766 µm^3^ (down by 19%) for 410 mOsm (Figure 2E). As these data were based on 2D measurements in which a spherical shape was assumed, we performed 3D‐reconstructed fluorescence confocal microscopy to validate this assumption. To this end, chondrocytes were stained using FITC and the actual cell volume was quantified. This showed that a change in extracellular osmolarity from 330 to 400 mOsm was associated with a decrease in cell volume of ≈21%. Volumetric analyses using 3D reconstructed fluorescence microscopy further revealed that the nuclear volume consistently was decreased with an average of ≈25% (Figure 2F,G). Together, this demonstrates that the difference between extracellular osmolarity between healthy and OA causes a considerable change in chondrocyte volume, which corroborates with our observed difference in size between chondrocytes in native cartilage of healthy or OA joints.

### 2.3 Microenvironmental Osmolarity Controls Intracellular Molecular Crowding via Cell Volume Alterations

**Figure 3:**
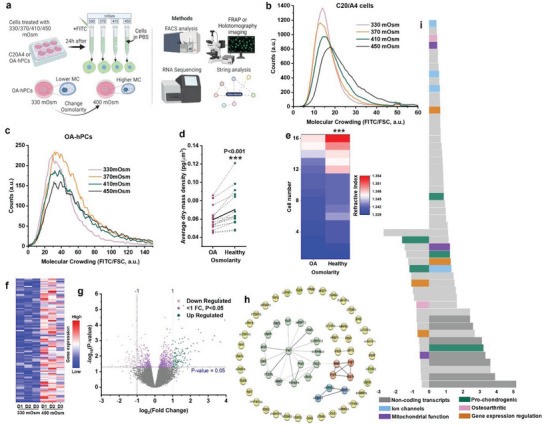
Osmolarity regulates molecular crowding in hPCs with minimal impact on chondrogenic gene expression. a) Schematic diagram showing that molecular crowding changes within cells as the cell size is controlled by osmolarity and the methods used to investigate the changes to molecular crowding. Flow cytometry analysis of molecular crowding in b) C20/A4 and c) OA hPCs after culturing them in 330–450 mOsm media for 24 h, *n* > 5000 cells, per condition. d) Average dry‐mass density of same OA hPCs exposed to OA and healthy‐like osmolarities (330 and 400 mOsm, respectively) simultaneously. Black line in the middle indicates the mean. Measured using holo‐tomography microscopy, 3 donors, *n* = 16 cells. e) Refractive index of a same OA hPC cell at 330 and 400 mOsm as measured by holotomography microscopy. 3 OA donors, *n* = 16 cells. f) heat‐map shows top‐100 differentially expressed genes (DEGs) in hPCs exposed to 400 mOsm as compared 330 mOsm. *N* = 3 OA and 3 healthy donors. g) volcano plot showing DEGs in hPCs exposed to 400 mOsm as compared 330 mOsm, *n* = 3 donors, per condition. h) STRINGdb network analysis shows biological processes impacted by osmolarity, colors indicate an individual network. i) stacked bar diagram showing top 100 DEGs in 400 mOsm as compared to 330 mOsm. Statistics: d,e) Paired t‐test, ***p < 0.001.

We next investigated whether intracellular molecular crowding was affected by the osmolarity‐induced change in cell volume (**Figure** **3**A). To measure the changes in intracellular molecular crowding, we developed a flow cytometry‐based quantification method that we termed FluoCrowd. Specifically, we stained virtually all cellular proteins using FITC (see Experimental Section) and measured forward scatter (FSC) and FITC fluorescence values in at least 5000 cells per condition. Intracellular molecular crowding was calculated by dividing the total FITC fluorescence value by the FSC value for each cell, which reveals a cellular protein content per defined volume unit. The human chondrocyte cell line C20/A4 was selected owing to its cell volume stable nature under standard culture conditions and was exposed to different osmolarities (330, 370, 410, and 450 mOsm) for 24 h. FluoCrowd measurements revealed that intracellular molecular crowding directly correlated with higher osmolarities (Figure 3B). We subsequently demonstrated that the osmolarity had a similar consistent dose‐dependent effect on the hPC's level of intracellular molecular crowding, albeit to a lesser extent (Figure 3C). Furthermore, we were able to confirm these differences by quantifying the average dry mass density (DMD) and refractive index (RI) per cell using holotomographic microscopy. We exposed OA‐hPCs to OA‐like (e.g., 330 mOsm) and healthy‐like (400 mOsm) osmolarity, and measured DMD and RI in each condition within the same cell. DMD (Figure 3D) and RI (Figure 3E) were both increased in OA‐hPCs after exposing them to healthy‐like osmolarity, which corroborated the observed increased intracellular molecular crowding. Our data therefore demonstrated that a healthy‐like osmolarity associates with a higher intracellular molecular crowding than an OA‐like osmolarity.

### 2.4 Molecular Crowding Did Not Effectively Change Chondrogenic Gene Expression in Absence of Specific Stimuli

Next, we investigated the effect of intracellular molecular crowding in the absence of specific (e.g., chondrogenic or inflammatory) stimuli on the gene expression level using whole transcriptome‐wide RNA‐seq analysis. OA and healthy hPCs were exposed to OA‐like and healthy‐like osmolarities for 48 h after which total cellular RNA was isolated and sequenced. Although a paired analysis combined OA and healthy hPCs revealed a minor yet consistent difference in gene expression patterns between hPCs exposed to either OA or healthy‐like osmolarities (Figure 3F,G), STRINGdb network analysis of the RNA‐seq data visualized that these few changes mostly revolved around a small network of proteins involved in cell‐matrix interactions (Figure 3H). Moreover, the number (<250) and amplitude (<5 fold) of differentially expressed genes were surprisingly small given the substantial change in osmolarity (Figure 3I and Data S1, Supporting Information). Although changes were observed for genes relating to mitochondrial function transcription and translation, pro‐chondrogenic and OA due to osmotic changes, pathway enrichment analysis of differentially expressed genes confirmed that the shift in gene expression profiles was surprisingly small (Figure 3I). DEGs within OA or healthy hPCs that were exposed to OA or healthy‐like osmolarities showed a similar trend (Figures S1 and S2, Supporting Information). Consequently, although osmolarity potently changes cell volume and intracellular molecular crowding within 48 h, the gene expression finger print remained largely unaffected in the absence of specific cell function‐steering cues.

### 2.5 Molecular Crowding Facilitates the Cell's Response to Anabolic Cues

**Figure 4:**
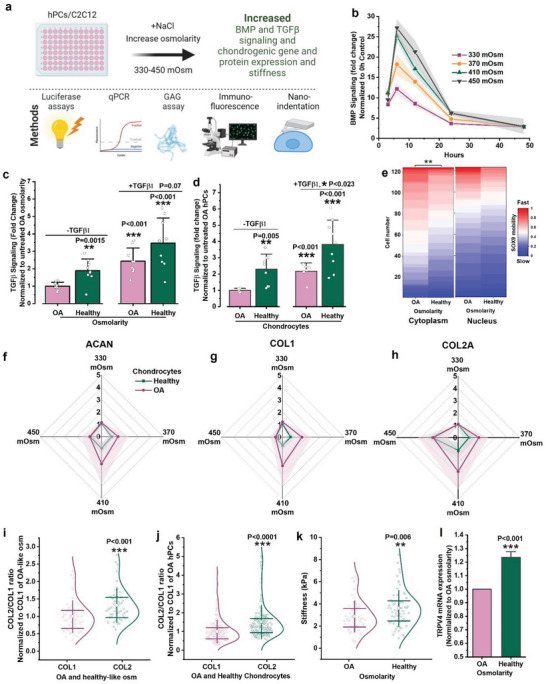
Osmolarity‐induced molecular crowding increases anabolic response. a) Schematic diagram showing cellular response to altered molecular crowding and the methods used to investigate the changes to cellular response. b) BMP signaling in C2C12 cells as measured by luciferase reporters after stimulating (BMP7, 10 ng mL^−1^) and culturing them in 330, 370, 410 and 450 mOsm at the indicated time points, *n* = 3 biological replicates. c) TGFβ signaling in OA hPCs after culturing them in OA or healthy‐like mOsm for 6 h, with or without TGFβ1 (10 ng mL^−1^) as measured by luciferase reporters, *n* = 3 donors and 3 biological replicates, per donor. d) TGFβ signaling in OA and healthy hPCs without changing osmolarity after 6 h of supplementing with or without TGFβ1 (10 ng mL^−1^) as measured by luciferase reporters, *n* = 2 OA and 3 healthy donors, 3 biological replicates per donor. e) FRAP measurements of SOX9‐mGFP(A76E) mobility in OA hPCs in cytoplasm and nucleus after exposing them to OA or healthy‐like osmolarities. 3 donors per condition, *n* > 120 cells, per condition (all donors combined). f) ACAN, g) COL1 and h) COL2A mRNA expression as measured by qPCR in OA and healthy hPCs exposed to 330, 370, 410, and 450 mOsm for 24 h, *n* = 3 donors. i) COL2/COL1 ratio as quantified by fluorescence intensity in OA hPCs after treating with either 330 or 400 mOsm for 5 d, *n* > 49 cells. j) COL2/COL1 ratio as quantified by fluorescence intensity in OA and healthy hPCs after culturing them in iso‐osmotic medium (330 mOsm) for 5 d, 3 donors per condition, *n* > 150 cells. k) Stiffness of OA hPCs exposed to OA or healthy‐like osmolarities for 24 h, 2 donors, *n* = 64 cells. l) TRPV4 gene expression in OA hPCs after exposing them to OA and healthy‐like osmolarity for 24 h. *n* = 3 donors. Statistics: c‐e,l) Unpaired t‐test, i–k) Mann‐Whitney U‐test, *p < 0.05, **p < 0.01, ***p < 0.001, shades and error bars b–d, f–h,l) indicate SD, whiskers and boxes in i–k) indicate SD and 25%, 75% percentile, respectively. All statistical significance is compared to normalized control, unless otherwise indicated.

We next investigated whether the osmolarity‐induced change in cellular volume and associated alteration of intracellular molecular crowding affect the cells’ ability to respond to anabolic cues (**Figure** **4**A). To this end, we measured the luciferase activity in genetically modified C2C12 cells which express a BMP reporter (BRE‐Luc). C2C12 cells were exposed to range of osmolarities (330, 370, 410, and 450 mOsm) as well as chondrogenic factor BMP7 (10 ng mL^−1^), and the luciferase activity was measured at 0 h, 3 h, 6 h, 12 h, 24 h, and 48 h postexposure. These measurements revealed that the cells’ ability to respond to BMP7 directly correlated with the osmolarity and hence intracellular molecular crowding. Cells in an OA‐like osmolarity responded the weakest and cells in a healthy‐like osmolarity responded the strongest to the same quantity of BMP7 (Figure 4B). This observation of increased sensitivity to an anabolic stimulus was in line with results from hPCs transfected with TGFβ reporter. In the presence but even in the absence of TGFβ1, both OA as well as healthy hPCs demonstrated a stronger luciferase expression upon crowding by changing the environmental osmolarity (Figure 4C). The only difference being that on average more crowded healthy chondrocytes expressed higher levels of TGFβ‐reporter than the on average less crowded OA chondrocytes (Figure 4D). To determine if the altered intracellular molecular crowding would have molecular and functional consequences, we investigated the effects on the intracellular speed of protein mobility, which governs intracellular signal transduction, protein‐protein and protein‐DNA interactions.^[^ ^29^ ^]^ For this, Transcription factor‐Fluorescence Recovery After Photobleaching (TF‐FRAP,^[^ ^30^ ^]^) was used to measure the mobility of the chondrogenic master transcription factor SOX9. Specifically, the mobility of transfected SOX9(A76E)‐mGFP^[^ ^30^ ^]^ was measured in the cytoplasm and nucleus of OA hPCs that were cultured under either 330 mOsm (e.g., OA‐like osmolarity) or 400 mOsm (e.g., healthy‐like osmolarity). This revealed that SOX9 protein mobility was detectably decreased in both cytoplasm and nucleus when the cells were exposed to a healthy‐like osmolarity as compared to an OA‐like osmolarity (Figure 4E), which indicates that transcription factor‐DNA binding is higher in hPCs exposed to healthy‐like osmolarity. To investigate whether this change was in part facilitated via gene expression, the mRNA levels of chondrogenic matrix markers (*ACAN*, *COL2A*, and *COL1*) were quantified in healthy and OA hPCs after culture in a range of osmolarities (330, 370, 410, and 450 mOsm) for 24 h. Interestingly, healthy chondrocytes were largely resilient to the osmotic changes as the expression of indicated genes did not change significantly. In contrast, OA hPCs were more sensitive to osmotic changes and expressed significantly higher mRNA levels of the chondrogenic maker genes at 410 mOsm (healthy‐like) osmolarity than at 330 mOsm (OA‐like) and 450 mOsm (supraphysiological; Figure 4F–H). Moreover, these results revealed that while OA chondrocytes exposed to distinct osmolarities in the absence of a specific stimulus do not change their gene expression profiles, the presence of an anabolic cue such as TGFβ results in a profound and significant effect on gene expression of OA chondrocytes when exposed to distinct microenvironmental osmolarities. In essence, this suggests that chondrocytes with different levels of intracellular crowding in an unstimulated microenvironment behave near‐identical, but when exposed to the same stimuli will offer significantly different responses.

To confirm that the observed changes in mRNA levels following exposure to an anabolic stimulus and distinct levels of microenvironmental osmolarity also translate to protein level changes, immunostaining was performed on OA hPCs after 5 d, which demonstrated that the COL2/COL1 ratio was significantly higher in OA‐hPCs cultured in a healthy‐like osmolarity compared to those cultured in an OA‐like osmolarity (Figure 4I). Notably, this effect of osmolarity resembled the altered COL2/COL1 ratio of OA hPCs and healthy hPCs (Figure 4J). GAG expression was also higher in hPCs exposed to healthy‐like osmolarity, however, statistical significance could not be demonstrated with the current experimental power (Figure S3, Supporting Information). Regardless, exposing OA hPCs to a healthy‐like osmolarity resulted in a higher Young's modulus that correlated with cellular stiffness compared to when being cultured in an OA‐like osmolarity (Figure 4K), which is another hallmark of a healthy chondrocyte phenotype.^[^ ^31^ ^]^ Gene and protein expression of ion channel TRPV4 increased in OA‐hPCs when exposed to a healthy‐like osmolarity (Figure 4L and Figure S4, Supporting Information) which is in line with previous study showing that a decrease in TRPV4 ion channel expression and activity promotes OA progression.^[^ ^32^ ^]^ Moreover, the cell proliferation ratio was significantly lower in OA hPCs exposed to a healthy‐like osmolarity (Figure S5, Supporting Information), which mimics the behavior of healthy hPCs, as their proliferation rate is substantially lower than that of OA hPCs.^[^ ^33^ ^]^ Together, these data support the hypothesis that osmolarity‐induced intracellular molecular crowding alters how a cell perceives its environment and impacts intracellular signaling processes at the protein level, especially when exposed to a chondrogenic stimulative cue such as TGFβ1. Moreover, the data suggests that intracellular molecular crowding could be a new key factor that can drive the chondrogenic potential and can be leveraged to renormalize OA hPCs into regaining an (at least partially) healthy phenotype.

### 2.6 Intracellular Molecular Crowding Decreases the Cellular Response to Catabolic Stimuli

**Figure 5:**
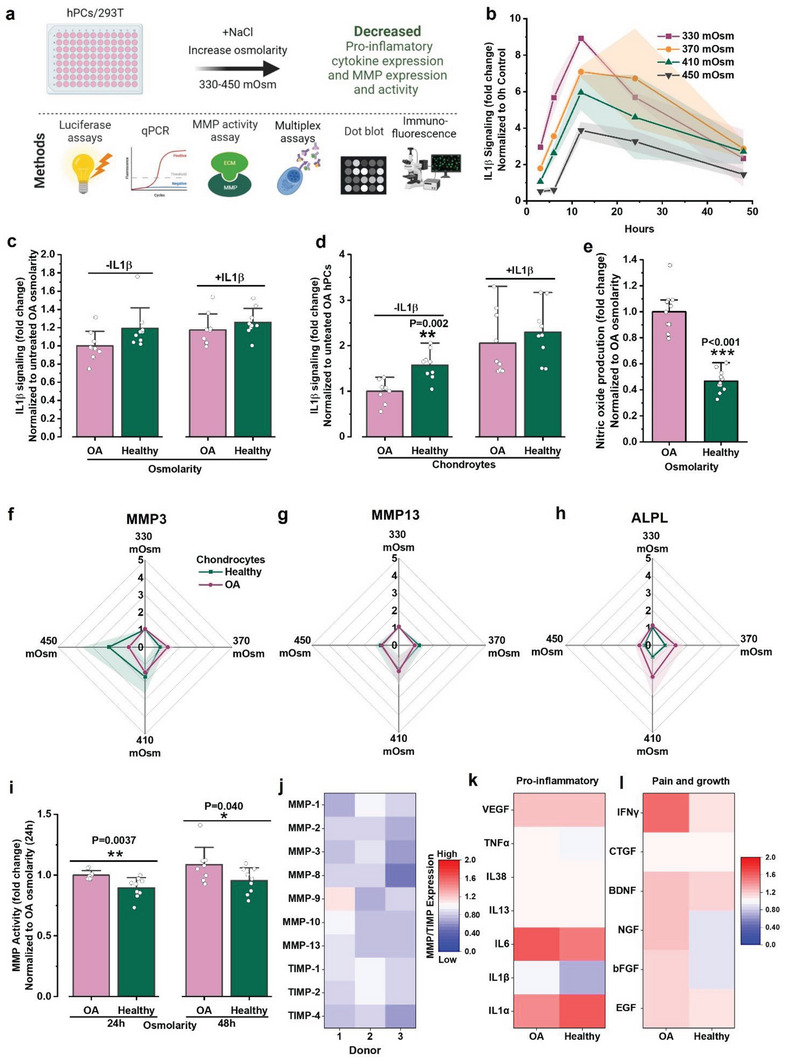
Osmolarity‐induced higher molecular crowding decrease catabolic signaling. a) Schematic diagram showing cellular response to altered molecular crowding and the methods used to investigate the changes to cellular response. b) IL1β signaling in HEK‐293T cells as measured by luciferase reporters after stimulating (IL1β, 10 pg mL^−1^) and culturing them in 330, 370, 410 and 450 mOsm at the indicated time points, *n* = 3 biological replicates). c) IL1β signaling as measured by luciferase reporters in OA hPCs exposed to OA and healthy‐like osmolarity, with or without IL1β (1 pg mL^−1^) for 6 h, *n* = 3 donors, 3 biological replicates. d) IL1β signaling as measured by luciferase reporters in OA and healthy hPCs cultured in iso‐osmotic medium (330 mOsm), with or without IL1β (10 pg mL^−1^) for 6 h, *n* = 3 donors, 3 biological replicates. e) Nitric oxide produced by OA hPCs after exposing them to OA and healthy‐like osmolarities for 24 h, *n* = 4 donors. f) MMP3, g) MMP13 and h) ALPL mRNA expression as measured by qPCR in OA and healthy hPCs exposed to 330, 370, 410 and 450 mOsm for 24 h, *n* = 3 donors. i) MMP activity in OA hPCs after treating them with OA and healthy‐like osmolarity for either 24 h or 48 h, *n* = 3 donors, 3 biological replicates per donor. j) Heat‐map showing MMPs and TIMPs expression as measured by dot blots in OA hPCs treated with OA or healthy‐like osmolarity for 48 h. Ratio of healthy/OA‐like (400/330) osmolarity is plotted in the heat‐maps, blue and red indicate lower and higher expression, respectively. *n* = 3 donors. Heat‐maps show multiplex analysis of expression of cytokines associated with k) pro‐inflammation and l) pain and growth pathways in OA and healthy hPCs after treating them with OA or healthy‐like osmolarities for 24 h. Ratio of healthy/OA‐like (400/330) osmolarity is plotted in the heat‐maps, *n* = 4 donors. Statistics: unpaired t‐test, *p < 0.05, **p < 0.01, ***p < 0.001, shades in b, f–h) and error bars in c–e,i) indicate SD. All statistical significance is compared to normalized control, unless otherwise indicated.

We next investigated whether osmolarity‐induced changes in intracellular molecular crowding also correlate with altered catabolic responses of hPCs (**Figure** **5**A). The inflammatory cytokine IL1β drives NF‐ĸB signaling, which drives a key catabolic process that orchestrates joint destruction during OA progression.^[^ ^34^ ^]^ To test whether intracellular molecular crowding changes IL1β signaling, we exposed HEK‐293T cells, which were genetically modified to express an NF‐ĸB luciferase reporter, to an environmental osmolarity of 330, 370, 410, or 450 mOsm as well as IL1β (10 pg mL^−1^) and measured the level of luciferase activity in a time resolved manner (Figure 5B). In contrast to the positive correlation of osmolarity on the anabolic response, NF‐kB activity was revealed to be not affected by osmolarity‐induced intracellular molecular crowding in OA hPCs (Figure 5C). Although NF‐kB reporter activity was higher in healthy hPCs compared to OA hPCs cultured in iso‐osmotic medium without IL1β, the increase in the reporter activity was not significantly different between OA and healthy hPCs in the presence of IL1β (Figure 5D). However, NF‐kB mediated nitric oxide production was lower in the OA hPCs exposed to healthy‐like osmolarity (Figure 5E). Moreover, gene expression analysis demonstrated that MMP3 and MMP13 mRNA levels were not significantly affected by the environmental osmolarity for healthy hPCs (Figure 5F,G). However, higher intracellular molecular crowding significantly decreased ALPL expression in healthy hPCs, while becoming higher in OA hPCs (Figure 5H). As our previous data had indicated that the osmolarity and subsequent change in intracellular crowding changed cells more on functional protein activity level than on mRNA level, we investigated the catabolic activity of OA hPCs exposed to either a healthy‐like (400 mOsm) or an OA‐like (330 mOsm, lower crowding) osmolarity for 24 h and 48 h. This revealed that MMP activity of OA hPCs in a healthy‐like osmolarity is lower than when cultured in an OA‐like osmolarity (Figure 5I). Analysis of the supernatant of these cells using dot blots demonstrated that expression levels of MMP1, MMP2, MMP3, MMP8, MMP9, MMP10, MMP13, TIMP1, TIMP3, and TIMP4 enzymes were all lower when OA hPCs were cultured in a healthy‐like osmolarity as compared to an OA‐like osmolarity (Figure 5J). Furthermore, culturing OA hPCs in a healthy‐like osmolarity also lowered the cells’ endoplasmic reticulum stress, (Figure S6, Supporting Information), which typically is upregulated during OA progression.^[^ ^35^ ^]^ Importantly, these data together suggest that the environmental osmolarity could potentially be used to partially renormalize the behavioral phenotype of OA hPCs thus potentially identifying a novel therapeutic avenue for the treatment of OA.

To further explore the potential use of the environmental osmolarity as a therapeutic modality for OA, we measured the effect of osmolarity on the expression of cytokines linked to inflammation, pain, and growth (elongation) in articular joints. Multiplex measurements of the supernatant media revealed that the expression of the pro‐inflammatory cytokine IL1β was lower in both OA and healthy hPCs when exposed to healthy‐like osmolarity while VEGF, IL6 and IL1α were increased (Figure 5K). Interestingly, a mild increase in the expression of cytokines linked to joint pain and growth (e.g., EGF, bFGF, NGF BDNG, CTGF and IFNγ) in hPCs was found upon exposure to a healthy‐like osmolarity (Figure 5L). Interestingly, these markers were also typically expressed at higher levels in OA hPCs versus healthy hPCs. Together, this data potentially suggests that osmolarity plays a role in joint pain sensation. Indeed, lavage treatment of the synovial cavity using higher osmolarity saline solutions is associated with reduced pain in patients.^[^ ^36^ ^]^

### 2.7 Energy Metabolism Shifts toward Glycolysis in hPCs with Higher Molecular Crowding

**Figure 6:**
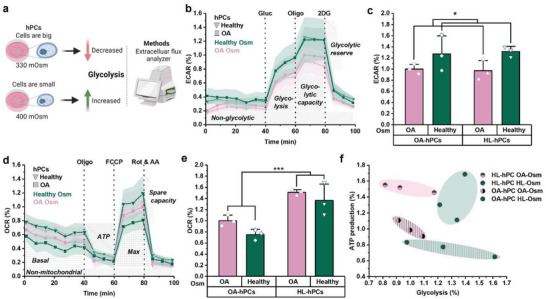
hPCs with higher molecular crowding source their energy through glycolysis. a) Schematic showing the change in glycolysis in response to altered molecular crowding which was assessed with a metabolic flux analyzer (Seahorse XFe96). b) Extracellular acidification rate (ECAR) profile of the glycolysis stress test, c) Glycolysis (based on glycolysis stress test) d) Oxygen consumption rate (OCR) profile of the mitochondria stress test, e) Mitochondrial ATP‐production (based on mitochondria stress test), f) Comparing mitochondrial ATP production (mitochondria stress test) with glycolysis (glycolysis stress test) for healthy and OA hPCs in healthy or OA‐like osmolarity, Parameters are calculated from the metabolic profiling, and normalized to the OA hPCs in OA‐like osmolarity. *n* = 3 donors per condition, statistics: two‐way ANOVA, *p < 0.05, **p < 0.01, ***p < 0.001, shades in b and d and error bars in c and e indicate SD.

Chondrocyte metabolism is known to be altered in OA.^[^ ^37^ ^]^ During OA progression, catabolism increases over anabolism^[^ ^37^ ^]^ and molecular crowding is known to influence metabolism.^[^ ^38^ ^]^ Moreover, it is known from other cell types that extracellular osmolarity dictates mitochondrial function via increased intracellular molecular crowding within the mitochondria.^[^ ^39^ ^]^ Glycolysis is known to be the primary energy source of chondrocytes.^[^ ^37^ ^]^ However, little is known regarding the osmolarity‐driven (de)crowding of hPCs toward their energy metabolism. We therefore investigated the metabolism in healthy and OA hPCs that were exposed to either an OA or healthy‐like osmolarity (**Figure** **6**A). The cells cultured in medium with a healthy‐like osmolarity displayed a significantly higher glycolysis‐linked extracellular acidification rate (Figure 6B,C) compared to those cultured in medium with a lower – OA‐like – osmolarity. Moreover, significant differences were observed in mitochondrial ATP production between healthy and OA hPCs (Figure 6E,F). Healthy cells had a significantly higher mitochondrial ATP‐linked respiration than OA cells. Notably, the basal respiration was significantly lower in OA hPCs compared to healthy hPCs (Figure S7, Supporting Information), indicating an overall lower mitochondrial respiration in OA hPCs. Moreover, the ATP‐linked mitochondrial respiration was lower in cells cultured in healthy‐like osmolarity. Taken together with the glycolysis results, these findings potentially suggest a shift in energy metabolism towards glycolysis in response to osmolarity‐induced higher molecular crowding. While the osmolarity induced higher intracellular molecular crowding could elevate glycolysis for healthy and OA hPCs, the reduced ATP production as observed in OA hPCs could not be restored.

## 3 Discussion

Understanding the pathology of osteoarthritis is the key to the development of a long sought disease‐modifying OA drug. Decades of research into OA pathology has revealed that it is a multifactorial disease that affects the entire joint in various ways, yet the OA pathology at the mechanistic molecular level is not yet fully understood. As such, attempts to development of disease‐modifying drugs for OA have largely restricted itself to the disease's symptoms rather than the disease itself. Here, we have identified osmolarity‐induced altered intracellular molecular crowding as a potential driver of OA pathology.

Molecular crowding is a key player in chondrocyte differentiation, cartilage development, and joint homeostasis.^[^ ^40^ ^]^ For example, during development, MSCs undergo condensation—in which cell size and volume decrease – prior to the differentiation into chondroprogenitors. These chondroprogenitors undergo series of differentiation processes, including terminal differentiation into either hypertrophic chondrocytes that mediate endochondral bone formation and mature articular chondrocytes that form permanent cartilage.^[^ ^40^, ^41^ ^]^ It is well established that during hypertrophic differentiation increases cell volume and adapts molecular crowding in a step‐manner, which is assumed to guide the differentiation process.^[^ ^42^ ^]^ As compared to hypertrophic chondrocytes, articular chondrocytes are significantly smaller in size and volume. However, during the progression of OA, articular chondrocytes are known to increase their size and volume.^[^ ^43^ ^]^ Moreover, this correlates with a decrease in the osmolarity of synovial fluid from ≈400 mOsm to ≈300 mOsm. However, the relationship between the altered osmolarity and volume in OA as well as the effect of OA on the molecular crowding of chondrocytes in affected joint has so far remained unknown.

In this study, we report that chondrocyte cell size is indeed smaller in healthy joints than OA joints and that these differences persist upon cell isolation. Moreover, flow cytometry and quantitative microscopic analysis revealed that the cellular volumes had an inverse relationship with the intracellular molecular crowding, which was thus higher in healthy chondrocytes. A key observation in this was that osmolarity induced intracellular molecular crowding altered chondrocyte function at the protein level. Specifically, increasing extracellular osmolarity to levels found in mimicking healthy joint results in a shift towards cartilage regeneration, which was substantiated by increased chondrocyte sensitivity to anabolic stimuli and reduced sensitivity to catabolic stimuli. Moreover, decreasing the extracellular osmolarity to levels found in OA joints had the opposite effect, and thus favored a catabolic response. We also demonstrated that increasing extracellular osmolarity resulted in renormalization of functional performance of OA hPCs toward healthy phenotype. In addition, we identified that these osmolarity‐driven alterations in cellular behavior were, at least in part, mechanistically orchestrated via altered intracellular molecular crowding. We thus here report on a novel cellular mechanism that controls a multitude of OA processes, which can be targeted to renormalize OA hPCs towards an anabolic phenotype, which offers novel opportunities for the design of a successful disease‐modifying OA drug.

Hyperosmolar irrigation solution is known to decrease pain and protect cartilage damage during arthroscopic procedures.^[^ ^20^, ^44^ ^]^ This correlates with our findings that chondrocytes exposed to healthy‐like osmolarity secrete comparatively lower pain‐associated cytokines. Moreover, cells exposed to healthy‐like osmolarity decreased NFĸB signaling, which is directly connected to pain and inflammation. However, the exact cellular mechanism by which molecular crowding mediates this process needs to be investigated. In addition, higher intracellular molecular crowding is known to favor protein‐protein and protein‐DNA interactions.^[^ ^45^ ^]^ Decreased mobility of chondrogenic factor SOX9 in cells that were exposed to healthy‐like osmolarity suggests that higher SOX9 binding might play a role in renormalizing OA hPCs to a healthy phenotype.

Previous studies targeting OA pathology have often proposed strategies to counter a single element of the disease by targeting a specific molecule.^[^ ^46^ ^]^ However, the multifactorial nature of the OA disease most likely requires a multi‐targeted approach to allow the treatment to address the distinct aspects of the disease. Targeting molecular crowding thus represents a unique approach as it affects chondrocytes’ functional intracellular signaling and sensitivity to microenvironmental cues at multiple levels. Importantly, luciferase based BMP7, IL1β, and TGFβ transcriptional reporters revealed that increased molecular crowding as found in healthy joints increased the chondrocytes’ responsiveness to the anabolic (pro‐regenerative) BMP7 and TGFβ signaling, while also having a decreased response to catabolic (pro‐inflammatory) NFĸB signaling. qPCR analysis corroborated these findings. Moreover, osmolarity‐induced higher molecular crowding shifted cellular energy metabolism towards glycolysis. Glycolysis is one of the favored energy metabolic pathways in chondrocytes.^[^ ^37^ ^]^ Molecular crowding could potentially realize these changes in metabolism via altered organellar organization, which was recently also been suggested in a recent study in which HeLa cells were exposed to Chloramphenicol.^[^ ^47^ ^]^ Thus, renormalizing OA hPCs with healthy‐like molecular crowding shifts its functional outcome from the disease state to healthy state. Together this suggests that intracellular crowding can play a pivotal role in normalizing or destabilizing a cell's anabolic, catabolic, and metabolic behavior and thus potentially can renormalize an OA‐affected chondrocyte in a multi‐level and multi‐target manner.

One of the possible mechanisms via which intracellular molecular crowding could potentially mediate these processes might be YAP/TAZ mechanotransducers. Recently, Yang et al. have reported that YAP/TAZ is translocated to the nucleus in chondrocytes cultured in 400 mOsm.^[^ ^48^ ^]^ Nuclear translocation of YAP/TAZ increased chondrogenic gene expression and decreased catabolic gene expression. In another study, Li et al. have shown that TAZ activity is essential for chondrocyte maturation and wound healing.^[^ ^49^ ^]^ Interestingly, YAP/TAZ activation is reported to attenuate NF‐κB signaling in OA chondrocytes.^[^ ^50^ ^]^ These emerging reports indicate a possible role for YAP/TAZ in the mechanistic regulation of the functional consequences of altered intracellular molecular crowding, and possibly vice versa.

It is of note that OA is a multifactorial disease, hence it is currently if our findings extrapolatable to OA in general or to specific patient subsets. Moreover, while the molecular crowding dictates cellular function in hPCs by modulating protein interactions, its role in the epigenetic modifications^[^ ^51^ ^]^ remains unknown. Furthermore, it cannot be excluded that the use of NaCl to adjust the extracellular osmolarity could introduce an experimental bias as other osmolytes are not included in this study. It is also noteworthy that although we identified a novel targetable mechanism to develop a DMOAD, currently, there is no well‐characterized library of drugs that could target this mechanism in a well‐understood manner, which challenges the immediate translation of our findings.

In summary, we identified osmolarity as a driver of OA pathology, and equally importantly, as a novel multi‐level mechanism for potential target for the development of a disease‐modifying drug. Specifically, the lower osmolarity levels in OA joints induce a shift towards cartilage destruction via decreased intracellular molecular crowding, which upon renormalization to healthy levels of molecular crowding reversed towards an anabolic phenotype that more closely resembled a healthy chondrocyte. We therefore report on a novel cellular mechanism that controls a multitude of OA processes, which can be targeted to renormalize OA hPCs towards an anabolic phenotype.

## 4 Experimental Section

### Osmolarity

Osmolarity of media/PBS was adjusted by adding NaCl_2_ and was measured with a Knauer K‐7400S osmometer (Knauer, Dortmund, Germany).

### Cell Culture and hPC Donors

C2C12 and C20/A4 culture media: DMEM (Invitrogen), with 10% FBS (Sigma). HEK‐293T culture media: MEM (Invitrogen) with 10% FBS (Sigma). Human primary chondrocyte (hPC) culture media: DMEM (Invitrogen), 10% FBS (Sigma), 20 × 10^−3^ m ascorbic acid‐2‐phosphate (Sigma) and non‐essential amino acids. Healthy hPCs were purchased (Articular Engineering), and OA hPCs were isolated from knee joints of patients that underwent knee replacement surgery following informed consent signed by each patient, which was performed under METC (2020‐7255) that was approved by Radboud UMC. Donor information such as age and gender is provided in Table S4 (Supporting Information). No major differences between the genders were observed in this limited dataset. All cells were cultured at in humidified incubators operating at 37 °C and 5% CO_2_.

### Luciferase‐Based Reporter Assays

C2C12 cells expressing BMP reporter (BRE‐luciferase)^[^ ^52^ ^]^ were seeded in 48‐well plates (15000 cells per well), and cultured in the presence or absence of BMP7 (10 ng mL^−1^; R&D systems, 354‐BP‐010). HEK‐293T cells expressing NFĸB Reporter^[^ ^53^ ^]^ were seeded in 48‐well plates (7000 cells per well), and cultured in the presence or absence of IL1β (10 pg mL^−1^, Peprotech, 200‐01B). These cells were cultured in 330, 370, 410, or 450 mOsm for 0 h, 3 h, 6 h, 12 h, 24 h, and 48 h. All measurements were performed in triplicate. hPCs were seeded in 96‐well plates (10 000 cells per well) and transfected with either NFĸB reporter plasmid^[^ ^54^ ^]^ or TGFβ reporter plasmid (SBE reporter^[^ ^55^ ^]^). The next day, the transfected chondrocytes were cultured in media with an osmolarity of either 330 or 400 mOsm, and with or without either IL1β (10 pg mL^−1^) or TGFβ1 (10 ng mL^−1^, R&D systems, 7754‐BH‐005) for 6 h. Transfection was performed using Lipofectamine LTX with Plus reagent (15338100, ThermoFisher) following the manufacturer's protocol. Luminescence was measured using Luminex 1420 (PerkinElmer). All measurements were performed in triplicate and repeated in three distinct OA donors.

### Protein Mobility Studies

OA hPCs were transfected with mutated SOX9‐mGFP(A76E), which does not have known binding sites in cytoplasm and weakly binds to DNA.^[^ ^30^ ^]^ Fluorescence recovery after photobleaching (FRAP) was performed as previously described.^[^ ^56^ ^]^ During imaging, cells were maintained in the Tyrode's buffer^[^ ^57^ ^]^ with either 330 or 400 mOsm. At least 40 cells were measured per condition for each of the three donors. Heatmaps were generated using OriginPro (2019b) software (OriginLabs). All measurements were performed for three OA donors (in >120 cells, all donors combined).

### Dot Blot (MMP/TIMP Expression)

OA hPCs were seeded in 6‐well plates (200 000 cells per well). The next day, culture media was refreshed with either 330 or 400 mOsm media. Supernatant media was collected after 48 h, and a dot blot assay was performed using the Abcam Human MMP Antibody Array–Membrane kit (ab134004) following the manufacturer's protocol. Chemiluminescence intensity values were calculated per dot using Fiji (ImageJ) and the 330/400 mOsm ratio per dot was plotted in a heatmap by OriginPro software. The assay was performed for three donors.

### MMP Activity Assay

OA hPCs were seeded in 12‐well plates (60 000 cells per well). The next day, media was refreshed with either 330 or 400 mOsm media, and the supernatant media was collected from separate wells after 24 h and 48 h. MMPs in the media supernatant were activated by 4‐aminophenylmercuric acetate (APMA) for 2 h. MMP activity assay was performed after 2 h of MMP activation. MMP Activity Assay Kit from Abcam (ab112146) was used following the manufacturer's protocol. Luminescence was measured using Luminex 1420 (PerkinElmer). All measurements were performed in triplicate for three OA donors.

### ER Stress Assay

OA hPCs were seeded in 96‐well plates (10 000 cells per well). The next day, media was refreshed with either 330 or 400 mOsm media. After 24 h, Thioflavin T (5 × 10^−6^ m, final concentration; 5 × 10^−3^ m stock prepared in 70% EtOH) was added to the cells and incubated for 10 min. Cells were imaged with a Zeiss LSM880 using a 20X/0.8NA objective and pinhole 0.85. Thioflavin T was excited with a 458 nm laser and the emission signal was detected at 480–520 nm. Total fluorescence intensity values were calculated per cell and the data was collected from more than 70 cells per condition for three donors.

### mRNA Isolation and qPCR

Healthy and OA hPCs were plated separately in 12‐well plates (50 000 cells per well). The next day, media was refreshed and cells were cultured in media with an osmolarity of 330, 370, 410, or 450 mOsm for 24 h. mRNA isolation and qPCR measurements were performed as described before.^[^ ^30^ ^]^ Gene expression is reported as relative mRNA expression normalized to untreated control and a stable housekeeping gene, RPL13. Primer sequences are specified in the supplementary information (Table S5, Supporting Information). All measurements were performed in triplicate for three healthy and three OA donors.

### Cell Proliferation Assay

OA hPCs were plated in 24‐well plates (10 000 cells per well). The next day, media was refreshed to either 330 or 400 mOsm media. After 8 d, cells were trypsinized and counted using a manual cell counter. From the total number of cells, 330/400 mOsm ratio was calculated. All measurements are performed in triplicate for three OA donors.

### FITC Staining

Trypsinized cells were washed with PBS (1x). FITC staining solution was prepared by adding 2.5 µL of FITC stock solution (20 mg mL^−1^ in ethanol) to 10 ml of osmolarity adjusted PBS. Cellular total protein was stained by resuspending the trypsinized cells in 1 mL of FITC staining solution and incubated for 10 min at 37 °C. Subsequently, cells were washed (3X) with osmolarity adjusted PBS.

### FluoCyte

Either hPCs or C20/A4 cells were plated in 6‐well plates (approximately 150 000 cells per well). The next day, culture media was replaced with osmolarity adjusted media (330, 370, 410, or 450 mOsm) in four different wells. After 24 h of replacing the osmolarity adjusted media, total cellular proteins were stained using FITC as mentioned above. Cells were run through a FACS machine (BD Aria II, BD Biosciences), excited with 488 nm laser, and forward scatter (FSC) and FITC fluorescence intensity were measured. Intracellular molecular crowding was calculated by dividing FITC florescence values by FSC values and multiplying with either 100 (for C20/A4 cells) or 10 000 (for hPCs). Shifting of histograms towards right indicates an increase in the intracellular molecular crowding. Measurements were performed in at least three distinct OA and healthy hPC donors.

### Confocal Microscopy

Total intracellular proteins in OA hPCs were stained using FITC as described above. Cells were diluted to low cell density (≈100 cells/10 µL) in hPC culture media with 330 mOsm. 10 µL of cell suspension was placed in a 24‐well plate. For 3D imaging, Z‐stack images of FITC stained cells suspended in PBS were recorded using a Nikon A1 confocal microscope with 20X/0.75NA objective and 488 nm laser with 1.65 µm slice thickness covering the entire cell. 1 µL of high osmolarity media was added to the cells in the 24‐well plate to increase the media's osmolarity to 400 mOsm, without disturbing the position of the cells. After a few minutes (<5 min), another image of the FITC signal of the cells in the same confocal place was recorded. Imaris (Oxford Instruments, UK) software was used for 3D construction of images. For cell/nuclear volume calculations, cells suspended in PBS were imaged in brightfield mode and DRAQ5 (62251, ThermoScientific) stained nuclei were imaged using a Nikon A1 confocal microscope with 10X/0.3NA objective and 633 nm laser. Nuclear area and volume were calculated using ImageJ.

### Cell Volume Change and Time Lapse Imaging

Unstained hPCs were suspended in culture media (330 mOsm) in a low density (≈100 cells/10 µL). 10 µL of cell suspension was placed in a 24‐well plate and brightfield timelapse images were taken every 10 sec using a EVOS FL (Invitrogen, AMF4300) microscope with a 40X objective (Invitrogen). Timelapse imaging was started and media osmolarity was increased to 400 mOsm after 50 s, and the time‐resolved imaging was continued for another 50 s. Size of a cell throughout the timelapse was calculated using ImageJ software. FITC stained C20/A4 cells suspended in osmolarity adjusted PBS (330, 370, or 410 mOsm) were imaged using a EVOS microscope (20X objective). Measurements were performed in >100 cells per condition.

### Collagen Staining (Immunofluorescence)

hPCs grown on glass coverslips were exposed to either 330 or 400 mOsm for 5 d and fixated with 10% formalin and immunostaining was performed as described before.^[^ ^58^ ^]^ Cells were imaged using a Zeiss LSM880 confocal microscope with a 20x/0.8 NA water immersion objective. Fluorescence intensity per cell was calculated using ImageJ.

### Nitric Oxide Measurement

OA hPCs were plated in a 24‐well plate (50 000 cells per well). The next day, the media was changed to either 330 or 400 mOsm, and incubated for 24 h. Subsequently, supernatant media was collected and nitric oxide measurements were performed using Griess assay kits (Promega, G2930). All measurements were performed in triplicate using four OA donors.

### GAG Quantification

hPCs were plated on a 24‐well plate (40000 cells per well). The next day, culture media was replaced with osmolarity adjusted media (either 330 or 400 mOsm) with or without TGFβ1 (10 ng mL^−1^, R&D systems, 7754‐BH‐005). After 96 h of replacing the media, supernatant was collected, and glycosaminoglycan content was quantified using dimethylmethylene blue (DMMB) assay as described before.^[^ ^59^ ^]^ All measurements were performed in duplicate and repeated in three OA donors.

### Nano Indentation

hPCs were plated on a 24‐well plate (25 000 cells per well). Next day, culture media was replaced with osmolarity adjusted media (either 330 or 400 mOsm). The Young's modulus of cells was measured using an interferometry‐based nano‐indenter (Pavone, optics11life) after 24 h of osmolarity treatment. The cells were indented with a probe with a 3 µm radius tip and a cantilever stiffness of 0.026 N m^−1^. The Young's modulus was obtained by fitting to an indentation depth of 300 nm with a Hertzian contact model (assuming a Poisson ratio of 0.5). All measurements were performed in at least 100 cells, collectively three OA donors.

### Histological Staining

Tissues were fixed in 4% w/v paraformaldehyde/10% neutral buffered formalin, decalcified in 12.5% w/v EDTA pH 8.0 and embedded in paraffin. After embedding 5 µm sections were mounted on to a glass slide and were deparaffinized by incubating in xylene (Klinipath, 4055‐9005) for 5 min (2x) and rinsed with 100% ethanol (Assink Chemie, 0050.41.210.5). H&E staining was performed following standard protocol. Stained tissue sections were imaged using a Nanozoomer using a 60x objective (Hamamatsu).

### Metabolic Assays

Approximately 24 h prior to the measurement of cell metabolisms, hPCs of six different donors (three healthy donors and three OA donors) were seeded in a Seahorse XF96 microplate (Agilent, 101085‐004) at a seeding density of 8000 cells per well. Prior to the measurements with a metabolic flux analyzer (Seahorse Bioscience, North Billerica, MA 24XP and/or 96XP), chondrocyte culture medium was replaced with assay medium consisting of RPMI medium (Sigma, R1383) containing 2 × 10^−3^ m GlutaMax (Gibco, 35050‐061) and 5% fetal bovine serum (Sigma, S0615). For the Mito stress test, 10 × 10^−3^ m glucose (Gibco, 15023021) was additionally added. Medium osmolarity was adjusted to either 300 mOsm or 400 mOsm using NaCl (S3014, Sigma). Metabolic assays were performed according to manufacturer's instructions, taking along four replicates per condition. Briefly, during the Mito stress test, oligomycin (1 × 10^−6^ m, Cayman Chemical, 11342), FCCP (3 µM, Sigma, C2920), and rotenone (1 × 10^−6^ m, Sigma, R8875) combined with antimycin (1 × 10^−6^ m, Sigma, R8875) were injected. During the Glycolytic stress test, glucose (10 mM), oligomycin (1 µM) and 2‐DG (10 mM, Sigma, D8375) were injected. Extracellular acidification rate (ECAR) and the oxygen consumption rate (OCR) were measured in mpH per min and pmol O_2_ per min respectively. After each injection, three measurements were taken. Data was normalized for cell numbers based on DNA quantity (QuantiFluor dsDNA system, Promega, E2670).

### Holotomographic Microscopy

hPCs were seeded in ibiTreat µ‐Dishes (Ibidi, 80136) at ≈60% confluency (40 000 cells per well). The next day, medium osmolarity was changed to either 330 or 400 mOsm. 24 h after changing osmolarity, 3D images of individual cells were collected using a holotomographic microscope (3D Cell Explorer‐fluo, Nanolive) with a 60X objective. For the measurement of changes in molecular crowding within the same cell, OA hPCs were first imaged in 330 mOsm after which medium osmolarity was increased to 400 mOsm and same cell was imaged within 5–10 min. 3D images were analyzed using EVE analytics (Nanolive) software and cell metrics such as refractive index and cell dry weight were calculated. All measurements were performed in at least three OA and healthy donors.

### Multiplex Assays

hPCs were seeded in a 24‐well plate (40 000 cell per well) in the hPC culture medium. The next day, cells were washed 3x and replaced with hPC culture medium without FBS and the osmolarity was adjusted to 330 or 400 mOsm. Supernatant was collected after 24 h of refreshing media. Samples were stored in −80 °C until multiplex analysis. Multiplex assays were performed using Luminex FLEXMAP 3D machine (Luminex corporation, USA). All measurements were performed in four OA and healthy donors.

### RNA‐Seq Analysis

Healthy and OA hPCs were plated separately on a six‐well plate (200 000 cells per well). Next day, medium osmolarity was changed to either 330 or 400 mOsm and maintained for 48 h. Subsequently, cells were lysed and mRNA was isolated using NucleoSpin RNA isolation kit (Macherey‐Nagel). Purity and concentration of RNA samples were measured using a Nanodrop 2000. RNA sequencing library was prepared using a QuantSeq 3′ mRNA‐Seq library prep kit (Lexogen, Vienna, Austria). Sequencing was performed at 2 × 150 bp on a NovaSeq 6000 (Illumina, San Diego, CA, USA). Raw sequence reads were aligned to the hg38 human reference genome using STAR (version 2.7.9a)^[^ ^60^ ^]^ using basic 2‐pass mode, using only one end of the pair due to the QuantSeq chemistry. Duplicate reads were marked using the Genome Analysis Toolkit (version 4.2.4.0).^[^ ^61^ ^]^ Reads were counted per exon in the GENCODE v38 human gene annotation using feature Counts (subread version 2.0.3).^[^ ^62^ ^]^ Read count normalization was performed using edgeR, and outlier detection was done by hierarchical clustering and plotting principal components. All measurements were performed in three OA and healthy donors. Top‐100 up and down regulated genes in OA and healthy hPCs that were exposed to healthy‐like osmolarity (as compared to OA‐like osmolarity) and corresponding volcano plots were determined (Data S2 and S3, Supporting information), respectively. The list of up and down regulated genes along with fold change (log 2) and p‐values (log 10) are given in a supplementary excel sheet (Data S4–S6, Supporting Information).

### Statistical Analysis

Statistical analyses were performed using OriginPro software. Unless otherwise indicated, the statistical significance is compared with respective normalized control. Type of statistics used per data is indicated in the figure legend. Statistical analysis of RNA‐seq data was performed using edgeR's exact‐test, followed by Benjamini‐Hochberg multiple testing correction within each comparison.

## Conflict of Interest

The authors declare no conflict of interest.

## Author Contributions

Conceptualization: K.G., J.P., and J.L.; Methodology: J.L., K.G., J.P., J.v.d.B., Y.R., A.v.W.; Investigation: K.G., M.M., M.B., and J.v.R.; Visualization: K.G., M.M., and J.L.; Supervision: J.P., and J.L.; Writing original draft: K.G.; Writing review & editing: K.G., Y.R., J.vd.B., J.P., and J.L.

## Supporting information

Supporting Information

Supporting Data 1

Supporting Data 2

Supporting Data 3

Supporting Data 4

Supporting Data 5

Supporting Data 6

## Data Availability

The data that support the findings of this study are available from the corresponding author upon reasonable request.
