# Supplementary material for: Osmolarity‐Induced Altered Intracellular Molecular Crowding Drives Osteoarthritis Pathology
Source: Adv Sci (Weinh). 2024 Jan 11;11(11):2306722. doi: 10.1002/advs.202306722 (PMC10953583; doi:10.1002/advs.202306722)
Supplement: Supplementary file 1 — Supporting Information [file ADVS-11-2306722-s006.pdf]

ADVANCED SCIENCE
Open Access

Supporting Information

for *Adv. Sci.*, DOI 10.1002/advs.202306722

Osmolarity-Induced Altered Intracellular Molecular Crowding Drives Osteoarthritis Pathology

*Kannan Govindaraj, Marieke Meteling, Jeroen van Rooij, Malin Becker, Andre J. van Wijnen, Jeroen J. J. P. van den Beucken, Yolande F. M. Ramos, Joyce van Meurs, Janine N. Post and Jeroen Leijten**

Supplementary Materials for

Osmolarity-induced altered intracellular molecular crowding drives osteoarthritis pathology

Kannan Govindaraj *et al.*

*Corresponding author. Email: jeroen.leijten@utwente.nl

This PDF file includes:

Figs. S1 to S7
Tables S1 to S5

Other Supplementary Materials for this manuscript include the following:

Data S1 to S6

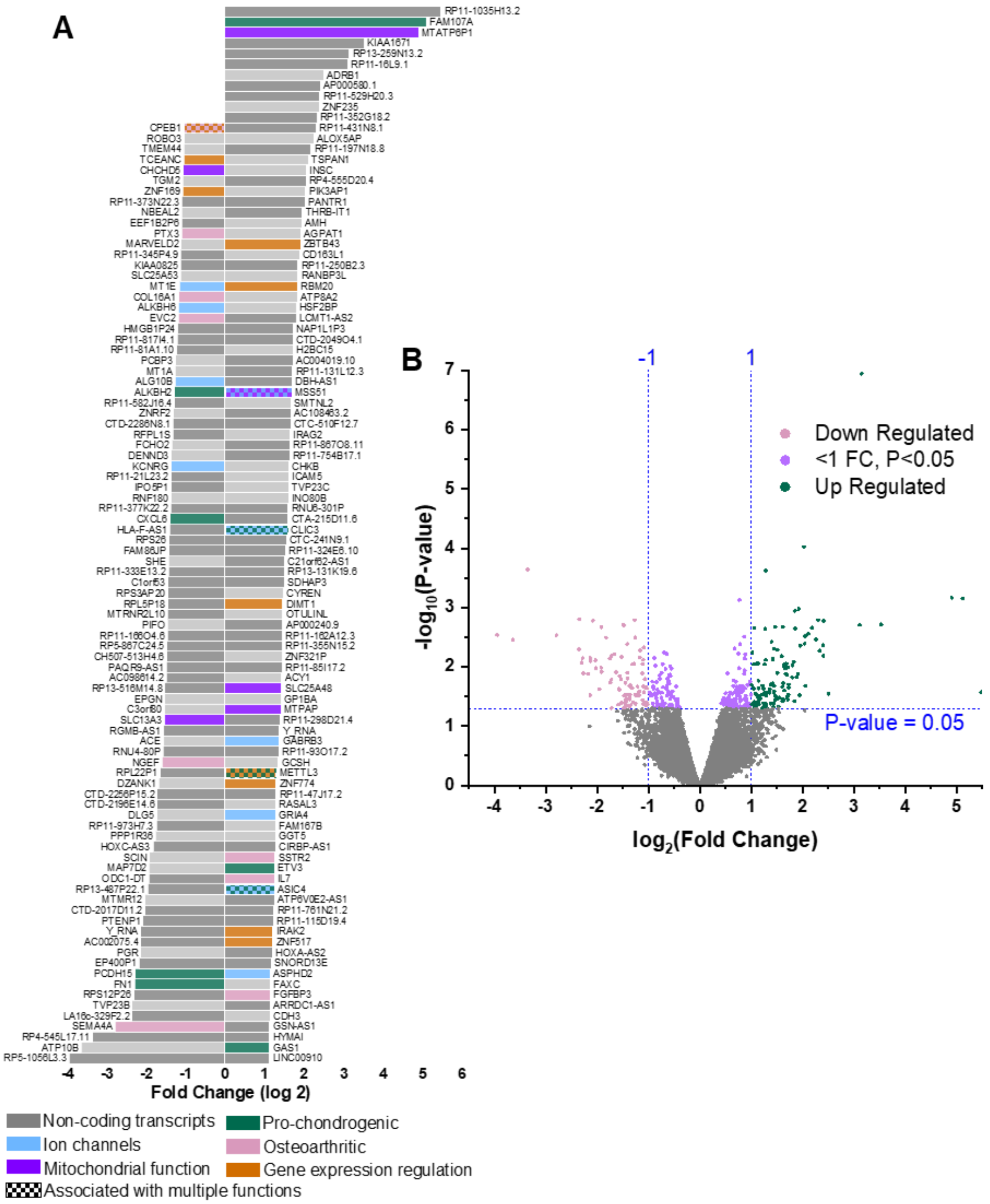

Fig. S1.

DEGs in OA hPCs that were exposed to healthy-like osmolarity. **(A)** Stacked-bar graph shows top-100 down and up-regulated genes in OA hPCs exposed to healthy-like osmolarity as compared to OA-like osmolarity. **(B),** volcano plot shows down (red), up (green) and significantly differently expressed (but <1 fold, purple) genes in OA hPCs exposed to healthy-like osmolarity.

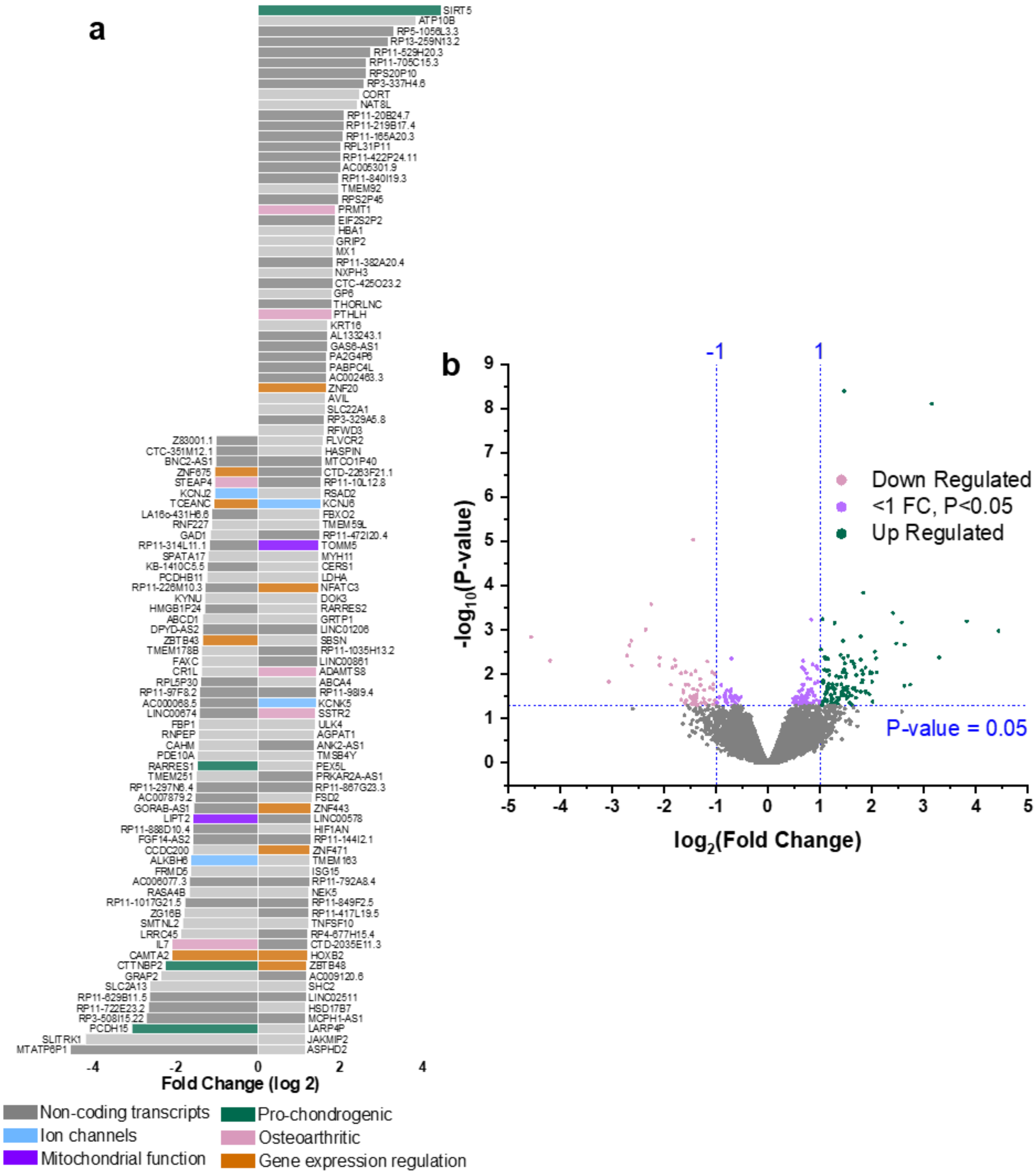

Fig. S2.

DEGs in healthy hPCs that were exposed to healthy-like osmolarity. **a,** Stacked-bar graph shows top-100 down and up-regulated genes in healthy hPCs exposed to healthy-like osmolarity as compared to OA-like osmolarity. **b,** volcano plot shows down (red), up (green) and significantly differently expressed (but <1 fold, purple) genes in healthy hPCs exposed to healthy-like osmolarity.

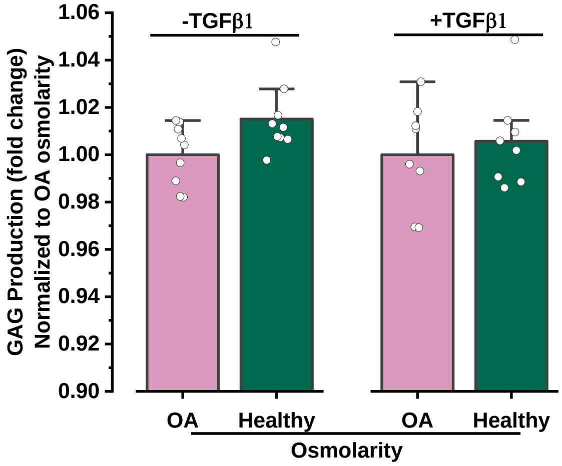

Fig. S3.
GAG produced by OA hPCs after 4 days of treatment with OA or healthy-like osmolarity and with or without TGFβ1 (10 ng/ml) as measured by DMMB assay, n = 3 OA donors, 2 biological replicates, per donor.

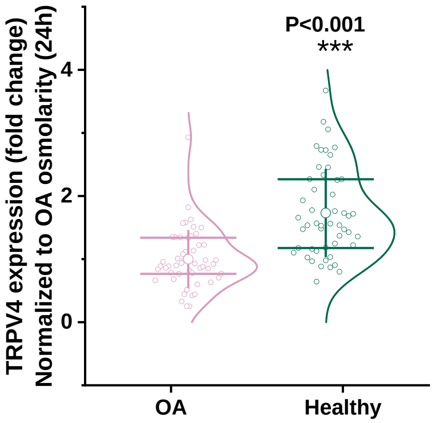

Fig. S4.

TRPV4 expression in OA hPCs exposed to OA and healthy-like osmolarities for 24 h. Fluorescence intensity values per cell were normalized to OA-like osmolarity, 1 donor, n = 51 cells, Statistics: t-test, unpaired, ***p<0.001.

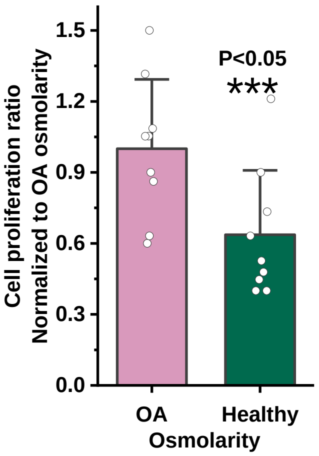

Fig. S5.
Proliferation ratio of OA hPCs as they were cultured in OA and healthy-like osmolarities for 8 days, n = 3 donors and 3 biological triplicates per donor. Statistics: t-test, unpaired, *$p<0.001$.

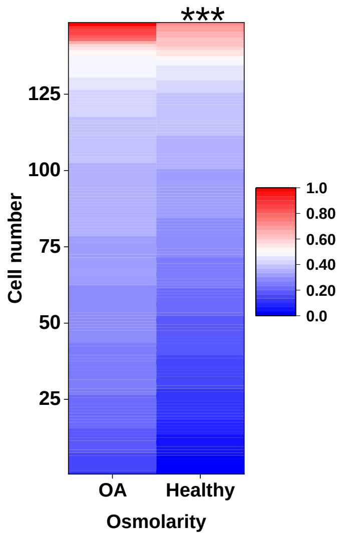

Fig. S6.

Heat-map showing ER stress levels in OA hPCs exposed to OA and healthy-like osmolarities, n = 2 donors, 148 cells. Statistics: t-test, unpaired, ***$p<0.001$.

Table S1

Literature reports on the osmolarity of articular tissue (OA = osteoarthritis, RA = Rheumatoid arthritis, SZ = Superficial Zone, DZ = Deep zone). Related to figure 2b.

Study	Normal (mOsm)	OA (mOsm)	RA (mOsm)	Ref
1	404 ±57	297 ±16.9	280±7.7	*(2)*
2	430			*(34)*
3	350-450			*(35)*
4	310-370, (SZ)<br>370-480 (DZ)			*(36)*
5	340-410			*(25)*
6	328.9± 41.3	295.6 ± 14.7	293 0± 7.2	*(37)*
7	383 ± 40			*(38)*
8		300	306	*(39)*
9		310.20 ± 5.54		*(40)*
10		281		*(41)*
11		300		*(19)*

Table S2

Our own measurements of osmolarity of Synovial fluid collected from OA knee joint. Related to figure 2b.

Donors	Gender	Age	Osmolarity (mOsm)
1	M	69	282
2	F	72	294
3	M	69	306
4	F	67	296
5	F	76	304
6	M	75	306
7	M	68	326
8	F	82	298
9	M	67	286
10	F	73	300
11	M	73	289
12	F	67	314
13	M	66	286
14	M	70	280

Table S3
Literature reports on effects of hypo or hyper osmolar irrigation solution in chondrocyte survival and function.

Study	OA/Healthy	Osmolarity (mOsm)	Description	Ref
1	OA	180, 280	Chondrocytes in cartilage explants released higher lactose dehydrogenase (LDH), higher indicating cell injury/damage.	26
	Healthy	380	Chondrocytes in cartilage explants released Lower LDH, indicating less cell injury/damage.	
2	OA	273	Post operative pain was higher in patients.	27
	Healthy	593	Post operative pain was lower in patients. Significantly less weight gain in the shoulder girth.	
3	OA	300	Higher expression of pro-inflammatory cytokines and MMPs by chondrocytes.	28
	Healthy	600	Lower expression of pro-inflammatory cytokines and MMPs by chondrocytes.	
4	OA	100, 255, 285	Higher chondrocyte cell death.	29
	Healthy	500, 600	Lower chondrocyte cell death.	
5	OA	180, 280	Higher chondrocyte cell death, higher elution of proteoglycans from cartilage explant, lower expression of type II collagen.	30
	Healthy	380, 580	Lower chondrocyte cell death, lower elution of proteoglycans from cartilage explant, higher expression of type II collagen.	
6	OA	285	Applying lower osmolarity irrigation solution resulted in a higher chondrocyte cell death at the injured site	31
	Healthy	600	Applying higher osmolarity irrigation solution resulted in a Lower chondrocyte cell death at the injured site	
7	OA	300	Expression of IL1β and IL-6 were higher in the cartilage tissue exposed to lower osmolarity.	32
	Healthy	400	Expression of IL1β and IL-6 were lower in the cartilage tissue exposed to lower osmolarity.	
8	OA	280	Lower TGFβ, BMP, COL2A expression	33
	Healthy	380	Higher TGFβ, BMP, COL2A expression	

Table S4
Details of donors used in this study.

Sl. No	Gender	Age	Health state	Lab reference
1	Female	48	Healthy	H-1461
2	Male	32	Healthy	H-1463
3	Male	48	Healthy	H-1466
4	Male	63	Healthy	H-1435
5	Female	73	Healthy	H-1441
6	Female	66	Healthy	H-1485
7	Female	70	OA	D22OA
8	Female	64	OA	D42OA
9	Male	61	OA	D67OA
10	Female	72	OA	D143OA
11	Female	66	OA	D122OA
12	Female	66	OA	D97OA
13	-	-	OA	D32OA
14	-	-	OA	D79OA

Table S5
Primer sequences.

Primer	F/R	Sequence
ACAN	For	5' AGGCAGCGTGATCCTTACC 3'
	Rev	5' GGCCTCTCCAGTCTCATTCTC 3'
COL1	For	5' GTCACCCACCGACCAAGAAACC 3'
	Rev	5' AAGTCCAGGCTGTCCAGGGATG 3'
COL2A	For	5' CCAGATGACCTTCCTACGCC 3'
	Rev	5' TTCAGGGCAGTGTACGTGAAC 3'
TRPV4	For	5' CCCGTGAGAACACCAAGTTT 3'
	Rev	5' TCACTCCAGGGCATTTCTTC 3'
MMP3	For	5' TGGCATTCAGTCCCTCTATGG 3'
	Rev	5' AGGACAAAGCAGGATCACAGTT 3'
MMP13	For	5' AAGGAGCATGGCGACTTCT 3'
	Rev	5' TGGCCCAGGAGGAAAAGC 3'
ALPL	For	5' ACAAGCACTCCCACTTCATC 3'
	Rev	5' TTCAGCTCGTACTGCATGTC 3'
RPL13	For	5’ AAAAAGCGGATGGTGGTTC 3’
	Rev	5’ CTTCCGGTAGTGGATCTTGG 3’
